# Assessing the reactivity to mobile phones and repeated surveys on reported care-seeking for common childhood illnesses in rural India

**DOI:** 10.7189/jogh.08.020807

**Published:** 2018-12

**Authors:** Harish Nair, Linda J Williams, Andrew Marsh, Pallavi Lele, Tathagata Bhattacharjee, Uddhavi Chavan, Siddhivinayak Hirve, Harry Campbell, Sanjay Juvekar

**Affiliations:** 1 Usher Institute of Population Health Sciences and Informatics, University of Edinburgh, Edinburgh, United Kingdom; 2 Institute for International Programs, Johns Hopkins University Bloomberg School of Public Health, Baltimore, Maryland, USA; 3 KEM Hospital Research Centre, Pune, India; 4 INDEPTH Network, East Legon, Accra, Ghana; * Joint senior authorship

## Abstract

**Background:**

Traditionally, health care-seeking for child illness is assessed through population-based and nationally representative demographic and health surveys (DHS) that are conducted once every five to seven years and are based on maternal recall. These maternal reports are subject to recall bias. Mobile phones (with the use of GPS technology) have the potential to constantly track movements of phone owners and provide high quality and more accurate data at a population level in low and middle income countries (LMICs) to assess the validity of maternal recall. We provided a group of mothers with smartphones installed with a location-aware application and visited them monthly to administer a survey questionnaire on care-seeking for diarrhoea, fever and cough with fever. This paper assesses for any reactivity to smartphones or repeated study contacts for measuring care-seeking and if this resulted in change in health care provider preference.

**Methods:**

We enrolled 749 mothers from rural areas of Pune district in Maharashtra, India and randomly allocated them to one of three groups – a longitudinal phone group, a longitudinal control group and a cross-sectional control group. We collected baseline information from mothers, including individual and household demographic and socio-economic characteristics and care-seeking preferences for child illness. We followed up both longitudinal groups monthly and each cross-sectional sub-group once over a period of 6 months. At each follow up, we administered questions identical to those in the National Family Health Survey (NFHS) questionnaire to determine an episode of diarrhoea, fever or cough within the last 15 days, care seeking for the same, and the type of provider. The data were analysed using the χ^2^ test or Fisher Exact Test for categorical variables, or with the Kruskall-Wallis non-parametric test for continuous variables (due to the non-normal nature of the data). Multivariable joint models of group and visit time were analysed with logistic regression methods.

**Results:**

All three groups were similar in their socio-demographic characteristics at baseline. We did not observe any significant difference in care seeking for diarrhoea, fever or cough with fever between groups. Also, we did not observe any significant difference in proportion of children seeking care from the private sector.

**Conclusions:**

We did not observe any reactivity in this study due to the presence of the phone (Hawthorne effect) or repeated study visits. The study also shows the potential of using GPS enabled smartphones to enrich DHS surveys in LMICs like India. However, further studies need to be conducted in other population groups before the findings can be generalised.

The World Health Organization estimates that globally about 5.6 million children younger than 5 years died in 2016 [1]. Pneumonia, diarrhoea and malaria are among the leading causes of mortality in under-5 children. More than half of child deaths worldwide are preventable using cost-effective interventions and improved access to health care [1]. However, there are many factors that contribute to the under-utilisation of effective health interventions in low- and middle-income countries (LMICs) [2]. Children are dependent on an adult carer for accessing health care and are therefore vulnerable to both demand side barriers (like recognition of symptoms, knowledge and attitudes, and affordability of the caregiver) as well as supply side barriers which are generally related to health systems [3].

Traditionally, health care-seeking behaviour for child illness in India is assessed through population-based and nationally representative demographic and health surveys such as the National Family Health Survey (NFHS). These surveys are conducted once every five to seven years and are based on maternal recall [4]. Maternal reports of care-seeking behaviour are subject to recall bias [5]. As mobile phone penetration continues to increase globally, recently reaching over 7 billion mobile connections worldwide [6], mhealth presents itself as a promising avenue through which to tackle challenges to collecting high quality and more accurate data at a population level [7]. With features such as GPS being available to constantly track movements of phone owners, mhealth-based interventions can be used to improve the measurement of health care-seeking in LMICs. The Improving Coverage Measurement (ICM) for Maternal, Newborn and Child Health Study in Pune involved development and deployment of an android mobile app (TrackCare) to record and transmit the mobile phone’s location data in real-time. This was done with a primary aim to validate maternal reporting of care-seeking for child illness on the a priori assumption that a mother’s movement to a health facility as determined by TrackCare was more accurate than her recall of a visit to a health facility. However, use of an app enabled smartphone where the participants are aware of their movements being monitored and repeated surveys (for validating recall) has the potential to alter health care seeking behaviour. This is known as Hawthorne effect and has been well described in studies reporting health-related behaviour [8]. Studies involving direct observation of or repeated contact with participants have reported significant change in behaviour [9,10], while those that have used devices monitoring participants remotely (eg, accelerometer) have either reported no effect or time-decaying effect [11,12]. The possible effect of altering a health-related behaviour as a result of exposure to a measurement device is called reactivity [12].

As a secondary aim of the larger ICM study, (in this paper) we aimed to assess the change in reported care-seeking subsequent to introducing the above-mentioned smartphone-based measurement tool and monthly face-to-face contact to administer a survey questionnaire on care-seeking for the three common childhood illnesses (diarrhoea, fever and cough with fever). We also wanted to examine if use of mobile phone and / or repeated contacts resulted in changing the health care provider preference (ie, if there was increased utilisation of private health care or vice versa).

## METHODS

We conducted the study in 22 villages in Pune district in Maharashtra State in the western region of India. The detailed methods are described elsewhere [13]. In summary, we randomly selected 926 mothers aged 15-49 years with at least one child under the age of five years for participation in the study. Of these, 749 mothers consented to participate in the study and were randomly allocated to one of the three groups – a longitudinal phone group (200 mothers), a longitudinal control group (100 mothers) and a cross-sectional control group (divided into six equal sub-groups of about 75 mothers each). Participants were enrolled between June and September 2015 and were followed up over six months. The mothers in the longitudinal phone group were given a smartphone with the TrackCare app, which was the measurement tool under study. We included a longitudinal control group to assess the potential bias in reporting care-seeking due to the presence of the study phone. We included the cross-sectional control group to determine whether changes in care-seeking reports were due to repeated administration of the survey questionnaire.

We collected baseline information from mothers including individual and household demographic and socio-economic characteristics, and care-seeking preferences for child illness. At each follow up, questions identical to those in the National Family Health Survey (NFHS) questionnaire were used to ask the mother to recall if the child had diarrhoea, fever or cough within the last 15 days, if care was sought, and the type of provider from which care was sought. Additional questions were asked to find when (how many days before the follow up) and where (name of health care facility) the care was sought.

### Statistical analyses

We calculated sample size only for the longitudinal phone group (as the primary objective was to test the validity of maternal recall) and this was based on the prevalence of diarrhoea, fever and cough under-5 children in the NFHS-3 [14]. The sample size for the longitudinal and cross-sectional control arms were based on what was feasible within the given resources (as assessing bias by the introduction of mobile phone tracking was a secondary objective). Our sample size of 200 mothers in phone group (assuming 2 under-5 children per mother) would have detected 80% of an estimated 480 episodes with 9% precision (Table S1 and S2 in **Online Supplementary Document(Online Supplementary Document)**).

The main outcome being tested during the monthly visit was care-seeking for childhood illness (as a binary variable). Some variables were compound variables, formed from the responses to two or more questions. We analysed any fever and fever with cough as two separate variables. We reduced the care provider categories to three groups (public health sector, private health sector and other). Private health sector included private practitioners qualified in allopathic medicine namely private doctor or clinic, private hospital and private paramedics. Similarly, we computed socio-economic status (analysed as wealth quintiles) as a composite of ownership of various assets (property, various durable goods, agricultural land, livestock, a bank/post office account, and health insurance or a health scheme), household building materials, drinking water source, toilet facility, and the presence of a servant or maid within the household [15].

The majority of the analyses examined the association between the group assignment or the visit time, with the various baseline and follow up variables. These were mainly cross-tabulations, analysed by either the χ^2^ test or Fisher Exact test, depending on the cell counts, or non-parametric comparisons of continuous data such as age (Kruskall-Wallis test). Wealth quintiles were analysed using the χ^2^ test for trend, since there is an ordinal relationship between the levels. The comparison of randomisation group and visit by care-seeking behaviour (“was care sought for the condition?”) were examined by simple logistic regression. Since we expected only a small proportion of the 2249 interviews would report diarrhoea, fever, and cough with fever, we assumed that including a model with parent random effect with nested child would risk over-fitting the data.

Due to the exploratory nature of the study, no adjustment for multiple testing was made. All analyses were conducted using Stata v12.1 (Stata Corp, College Station, TX, USA).

### Ethical considerations

Mothers provided written informed consent prior to enrolment and randomization. Prior to consent, mothers were informed that those assigned to the phone group would be allowed to keep the study phones even if they withdrew their participation at any stage of the study. The study was approved by the Ethics Committee of the KEM Hospital Research Centre, Pune, India (Study ID No. 1415) and the University of Edinburgh, UK. The study was not registered with the Clinical Trials Registry as it was not considered by investigators to meet the criteria for such a trial. Study groups differed in the method and frequency with which their care-seeking behaviour was measured but no group was provided with a health-related intervention intended to affect a health outcome.

## RESULTS

We enrolled and followed up 200 mothers in the intervention phone group and 100 mothers in the longitudinal control group at monthly intervals over six months (Table S3 in **Online Supplementary Document(Online Supplementary Document)**). We also enrolled 449 mothers in the cross-sectional control group, divided them up in six sub-groups (five with 75 mothers each and one with 74 mothers) and followed up each group once over the six-month period. We observed loss to follow up (increasing with time) in both the phone and control groups, with a greater proportion being lost to follow up in the control group (20% compared to 10.5% in the phone group).

We compared the mothers in the three groups for various baseline characteristics in both parents (number of children younger than five years, age, years of education, occupation, household wealth quintile, ownership of mobile phone and religion) and found no significant difference between the three groups (**Table 1**). There was some indication of a difference in wealth quintile between the groups (*P* = 0.016), with mothers in the cross-sectional group more likely to be in quintiles 1 and 2 than the phone or longitudinal control groups. We also observed a significant difference in the ownership of smartphones (at household level) in the three groups.

**Table 1:** Characteristics of the three groups compared at baseline*

	Phone group (n = 200)	Longitudinal control group (n = 100)	Cross-sectional control group (n = 449)	*P*-value
Number of children <5 y old to a mother:				0.74
1	87 (43.5%)	50 (50%)	220 (49%)	
2	104 (52%)	46 (46%)	209 (46.5%)	
3	9 (4.5%)	4 (4%)	20 (4.5%)	
Median maternal age [Median (IQR)], n*	25 (23-27), n = 194	25 (23-27), n = 99	25 (23-27), n = 442	0.96
Mother ever attended school (%)	193/193 (100%)	98/99 (99%)	436/442 (99%)	0.27
Completed years of maternal education (Median, IQR), n*	10 (9-12), n = 192	10 (9-12), n = 98	10 (9-12), n = 433	0.12
Mother’s occupation- agriculture (%)	37/60 (62%)	25/34 (74%)	94/134 (70%)	0.35
Father’s age (Median IQR), n*	30 (28-32), n = 193	30 (28-32), n = 98	30 (28-32), n = 437	0.88
Father’s completed years of education, Median (IQR), n*	12 (10-13), n = 189	10 (9-12), n = 97	11 (9-13), n = 432	0.26
Father’s occupation				0.10
Agriculture (%)	30/188 (16%)	12/98 (12%)	94/429 (22%)	0.10
Manufacturing (%)	91/188 (48%)	56/98 (57%)	202/429 (47%)	
Any mobile phone ownership in household	196/197 (99.5%)	98/98 (100%)	442/446 (99.1%)	1.00
Smartphone ownership in household	133/193 (68.9%)	45/98 (45.9%)	245/431 (56.8%)	0.0004
Household wealth quintile:				0.016
1	29 (15%)	20 (20%)	101 (22%)	
2	36 (18%)	17 (17%)	97 (22%)	
3	45 (23%)	22 (22%)	83 (18%)	
4	49 (25%)	24 (24%)	77 (17%)	
5	41 (21%)	17 (17%)	91 (20%)	
Religion of head of household- Hindu	184/199 (92%)	88/99 (89%)	410/448 (92%)	0.30

y – years, IQR – interquartile range

*Where there are missing data, the number of respondents have been given.

**Figure 1:**
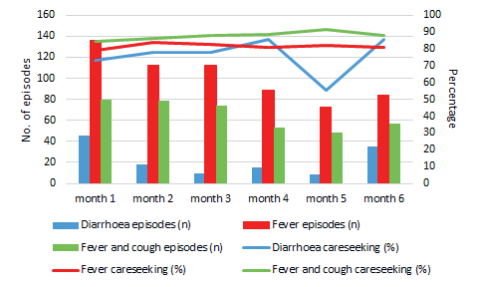
Episodes of diarrhoea, fever and cough with fever in under-5 children and care seeking for these by month during study period.

We identified 133 episodes of diarrhoea in children younger than five years in the three groups over the six-month period, 101 (76%; 95% CI = 69%-83%) of which sought care (Table 2). We did not observe any significant difference in care seeking for diarrhoea between visits (**Figure 1**, Table S4 in **Online Supplementary Document(Online Supplementary Document)**) or between groups (**Table 2**).

**Table 2:** Episodes of diarrhoea, fever and pneumonia in children younger than 5 y and care seeking in the three groups

	Phone group (n = 1588)	Longitudinal control group (n = 759)	Cross-sectional control group (n = 555)	*P*-value
Children <5 y with diarrhoea in past 2 weeks	64/ 1581 (4%)	42/757 (6%)	27/555 (5%)	
Proportion of children with diarrhoea who sought care (%, 95% CI)*	47/63 (75% [64%-85%])	33/41 (80% [68%-93%])	21/27 (78% [62%-93%])	0.69
Children <5 y with fever in past 2 weeks	341/1570 (22%)	154/758 (20%)	105/551 (19%)	
Proportion of children with fever who sought care (%, 95% CI)†	283/341 (83% [79%-87%])	120/151 (79% [73%-86%])	83/105 (79% [71-87])	0.51
From public sector	24 (8.4%)	6 (5%)	4 (5%)	
Number of days from onset of fever to first seeking care or advice (Median, IQR), n‡	0 (0-1), n = 280	0 (0-1), n = 118	0 (0-1), n = 83	
Children <5 y with fever and cough in past 2 weeks	224/1542 (14%)	95/747 (13%)	72/545 (13%)	
Proportion of children with fever and cough who sought care (%, 95% CI)§	195/221 (88% [84%-92%])	79/94 (84% [77%-91%])	64/72 (89% [82%-96%])	0.50
From public sector	17 (8.7%)	7 (8.9%)	2 (3.1%)	

y – years, IQR – interquartile range, CI – confidence interval

*χ^2^ test: *P* = 0.69.

†χ^2^ test: *P* = 0.51.

‡n = number of respondents.

§χ^2^ test: *P* = 0.50.

There were 606 episodes of fever (including 391 with cough) of which 486 (80%, 95% CI = 77%-83%) and 338 (86%, 95% CI = 83%-90%) respectively sought care (**Table 2**). We did not observe any significant difference in care seeking for fever between groups (**Table 2**) or between visits (**Figure 1**, and Table S4 in **Online Supplementary Document(Online Supplementary Document)**).

We did not observe any significant difference in proportion of children seeking care from the private sector, the interval between appearance of symptoms and care seeking, and the proportion of children with fever (with or without cough) who were administered medication for the illness across the three groups (**Table 2**).

## DISCUSSION

We enrolled and actively followed up 749 mothers in three groups (one that was provided a smartphone and followed up monthly, another only followed up monthly, and the third only once) over a six-month period. We did not observe any significant difference in the reported incidence or care seeking for diarrhoea, fever or fever with cough in under-5 children in the three groups. This suggests no reactivity resulting from to the awareness that movement was being monitored through the smartphone or due to repeated study contacts.

We observed that in the 724 under-five children in the study, fever was the most common illness (1.65 episodes per child per year). Care seeking varied by illness. For example, care seeking varied from about 78% in diarrhoea to about 87% in fever with cough. However, we did not observe any difference in the illness and choice of provider. For example, about 92% of the children were treated in the private sector for fever (with or without cough). The finding of low utilisation of public services is consistent with other published findings from Indian subcontinent. For example, a study from Bangladesh reported that 90% of children presenting with symptoms of diarrhoea for whom care was sought were taken to a private sector provider even though the public health care sector was well established in the country [16]. In Maharashtra, private health care has historically been seen to be “far superior” to government provided care, with women seeking care feeling that the benefits outweigh the cost [17]. Despite efforts over the last decade by the Indian government to increase use of public health care systems, private sector is still the preferred provider of health care [18]. This highlights that promotion of ineffective services is unlikely to increase utilisation of public health care by people unable to afford private care. Improvement of these health services must go hand in hand with promotion to increase behavioural change and reduce the health inequalities between the socioeconomic classes.

Our inability to observe any significant differences between the phone and control group, could be attributed to lack of power. Although, we calculated sample size for the phone group based on the combined prevalence of all the three conditions reported in literature, we were not sufficiently powered for some of the sub-group analyses. The lack of any significant differences between the phone and the longitudinal control group could also be attributable to either the phone having no effect, or a change in health seeking behaviour with repeated contacts affecting both groups equally. This finding is similar to what has been observed by Tobias and Inauen in Columbia where the intervention group (receiving solar water disinfection promotional campaign) and a control group were followed up through repeated contacts using face to face surveys [19]. One of the key limitation of our research design was that we were not well set up to answer if the repeated contacts influenced a change in health care seeking behaviour. Such an analysis would have required each cross-sectional group to be of the same size as the phone and longitudinal control group. However, it must be pointed out that Tobias and Inauen argue that repeated surveys at short interval when introducing a new intervention have only a minimal effect on behaviour if the face to face contacts are not performed more often than once a week [19]. In our context, mobile phone ownership is very high in India and >99% of the study participants had a mobile phone. Smartphone ownership was significantly different in the three groups – 46%-57% of the participants in the control groups had smartphones of their own (compared to 69% in the phone group) (**Table 1**). Therefore, it is unlikely that presence of a phone itself would alter behaviour. Also, the population in Vadu HDSS is accustomed to regular surveys (on a six-monthly basis) and have participated in clinical studies. Therefore, it is unlikely that an increased frequency of contacts would alter their behaviour. We trained the research team in administering the surveys meticulously, and supportive supervision with random spot checks was maintained throughout the study. By doing this, we attempted to minimise observer and interviewer bias.

This study shows potential of a ubiquitous technology like smartphones to enrich DHS surveys in LMICs like India. The early results are encouraging, but further studies need to be conducted in other population groups before the findings can be generalised.
