# Supplementary material for: Assessing the reactivity to mobile phones and repeated surveys on reported care-seeking for common childhood illnesses in rural India
Source: J Glob Health. 2018 Sep 19;8(2):020807. doi: 10.7189/jogh.08.020807 (PMC6150610; doi:10.7189/jogh.08.020807)
Supplement: Online Supplementary Document [file jogh-08-020807-s001.pdf]

Online Supplementary Document

Harish et al. Assessing the reactivity to mobile phones and repeated surveys on reported care-seeking for common childhood illnesses in rural India

J Glob Health 2018;8:020807

Supplementary table 1: Percent of children under 5 years of age with suspected illness and percent seeking treatment, by illness type (NFHS-3, 2005-2006)

	Percent with suspected illness	Percent seeking treatment
**Symptoms of ARI (i.e. cough and fast breathing)**	6.7%	72.4%
**Symptoms of malaria (i.e. fever)**	11.0%	83.9%
**Diarrhea**	8.1%	77.3%

*All indicators represent illness episodes and treatment occurring in the two weeks prior to the survey for children under 5 years of age.

We calculate the proportion of all children seeking care for a specific illness as the product of the two columns above. For example, we expect that 4.9% of all children will seek care for ARI in the two weeks before the survey (6.7% x 72.4% = 4.9%). The combined prevalence of care-seeking for all three illness combined is as:

(6.7% x 72.4%) + (11.0% x 83.9%) + (8.1% x 77.3%) = 20.3%

In other words, for every 100 children interviewed we would expect 20.3 to report a care-seeking event.

Supplementary table 2: Sample size required for given levels of precision and proportion of care-seeking events that are confirmed

	Base Prevalence			
**Precision**	50%	70%	75%	80%
**5%**	385	323	289	246
**6%**	267	225	201	171
**7%**	196	165	147	126
**8%**	151	127	113	97
**9%**	119	100	89	**76**
**10%**	97	81	73	62

Supplementary table 3: Number of mothers enrolled and followed up (by month) in the intervention (phone) and control groups

	Phone group (n=200)	Longitudinal Control Group (n=100)	Cross-sectional Control Group (n=449)					
			Grp1 (n=75)	Grp2 (n=75)	Grp3 (n=75)	Grp4 (n=74)	Grp5 (n=75)	Grp6 (n=75)
**Month 1**	191	97	71					
**Month 2**	191	95		70				
**Month 3**	183	93			65			
**Month 4**	180	87				66		
**Month 5**	174	83					61	
**Month 6**	179	80						60

Supplementary table 4: Episodes of diarrhoea, fever and pneumonia in under-5 children and care seeking for these by month during study period

	Month 1	Month 2	Month 3	Month 4	Month 5	Month 6
**Total episodes of diarrhoea (% [95%CI])**	46/498 (9.2% [6.8, 12.1%]	18/498 (3.6% [2.2, 5.7%])	10/482 (2.1% [1.0, 3.8%])	15/490 (3.1% [1.7, 5.0%])	9/459 (2.0% [0.9, 3.7%])	35/466 (7.5% [5.3, 10.3%])
**Episodes of diarrhoea seeking care (% [95%CI])[1]**	33/45 (73.3% [58.1, 85.4%])	14/18 (77.8% [52.4, 93.6%])	7/9 (77.8 [40.0, 97.2%])	12/14 (85.7% [57.2, 98.2%])	5/9 (55.6% [21.2, 86.3%])	30/35 (85.7% [69.7, 95.2%])
**Total episodes of fever (% [95%CI])**	136/495 (27.5% [23.6, 31.6%]	112/495 (22.6% [19.0, 26.6%]	112/482 (23.2 [19.5, 27.3%]	89/490 (18.2% [14.8, 21.9%]	73/454 (16.1 [12.8, 19.8%]	84/463 (18.1% [14.7, 22.0%]
**Episodes of fever seeking care (% [95%CI])[2]**	105/133 (78.9% [71.0, 85.5%])	92/110 (83.6% [75.4, 90.0%])	91/110 (82.7% [74.3, 89.3%])	71/88 (80.7% [70.9, 88.3%])	59/72 (81.9% [71.1, 90.0%)	68/84 (81.0%[70.9, 88.7%])
**Total episodes of fever and cough (% [95%CI])**	80/473 (16.9% [13.6, 20.6%])	79/485 (16.3% [13.1, 19.9%])	74/479 (15.4% [12.3, 19.0%])	53/488 (10.9% [8.2, 14.0%])	48/451 (10.6% [8.0, 13.9%])	57/458 (12.4% [9.6, 15.8%])
**Episodes of fever and cough seeking care (% [95%CI])[3]**	66/78 (84.6% [74.7, 91.8%]))	67/78 (85.9% [76.2, 92.7%])	65/74 (87.8% [78.2, 94.3%])	47/53 (88.7% [77.0, 95.7%])	43/47 (91.5% [79.6, 97.6%])	50/57 (87.7% [76.3, 94.9%])

[1] χ^2 test: p=0.47
[2] χ^2 test: p=0.95
[3] χ^2 test: p=0.90
